# Wound Healing Property of Curcuminoids as a Microcapsule-Incorporated Cream

**DOI:** 10.3390/pharmaceutics11050205

**Published:** 2019-05-01

**Authors:** Lee Fung Ang, Yusrida Darwis, Rhun Yian Koh, Kenny Voon Gah Leong, Mei Yeng Yew, Lip Yee Por, Mun Fei Yam

**Affiliations:** 1 School of Pharmaceutical Sciences, Universiti Sains Malaysia, Pulau Pinang 11800, Malaysia; ang.leef@gmail.com (L.F.A.); yusrida@gmail.com (Y.D.); 2 Pathology Department, International Medical University, Kuala Lumpur 57000, Malaysia; rhunyian_koh@imu.edu.my (R.Y.K.); kenny_voon@imu.edu.my (K.V.G.L.); nancyew_aries@hotmail.com (M.Y.Y.); 3 Faculty of Computer Science and Information Technology, Universiti Malaya, Kuala Lumpur 50603, Malaysia; porlip@um.edu.my

**Keywords:** wound healing, microencapsulation, curcuminoid

## Abstract

Curcuminoids have been used for the management of burns and wound healing in traditional Chinese medicine practices but the wide application of curcuminoids as a healing agent for wounds has always been a known problem due to their poor solubility, bioavailability, colour staining properties, as well as due to their intense photosensitivity and the need for further formulation approaches to maximise their various properties in order for them to considerably contribute towards the wound healing process. In the present study, a complex coacervation microencapsulation was used to encapsulate curcuminoids using gelatin B and chitosan. This study also focused on studying and confirming the potential of curcuminoids in a microencapsulated form as a wound healing agent. The potential of curcuminoids for wound management was evaluated using an in vitro human keratinocyte cell (HaCaT) model and the in vivo heater-inflicted burn wound model, providing evidence that the antioxidant activities of both forms of curcuminoids, encapsulated or not, are higher than those of butylated hydroxyanisole and butylated hydroxytoluene in trolox equivalent antioxidant capacity (TEAC) and (2,2-diphenyl-1-picryl-hydrazyl-hydrate) (DPPH) studies. However, curcuminoids did not have much impact towards cell migration and proliferation in comparison with the negative control in the in vitro HaCaT study. The micoencapsulation formulation was shown to significantly influence wound healing in terms of increasing the wound contraction rate, hydroxyproline synthesis, and greater epithelialisation, which in turn provides strong justification for the incorporation of the microencapsulated formulation of curcuminoids as a topical treatment for burns and wound healing management as it has the potential to act as a crucial wound healing agent in healthcare settings.

## 1. Introduction

A wound can be defined as a physical rupture at the epithelial integrity of the skin. While understanding the formations of wounds can be considerably straightforward, the wound healing process is a lengthy one and requires deep exploration. The wound healing process comprises of a series of overlapping phases, namely the inflammatory, proliferative, and remodeling phase [1]. The healing process often starts with the inflammation phase of the wound, with edema, erythema, heat, and pain as its accompanying characteristics. In the later stages of inflammation, macrophages play one of the major roles, responsible for the digestion of and cleaning the cellular debris from the wound. With the macrophages at work, the proliferative phase begins, which consists of the migration of fibroblasts, the deposition of the extracellular matrix, and the formation of the granulation tissue. The healing wound exhibits a moist, shiny, hyperaemic, and reddish appearance on the granulation tissue, however if the wound appears to have excessive inflammation, and soft and friable tissue with a beefy-red colour, it indicates that the wound is healing poorly. The remodeling phase is initiated concurrently with the development of granulation tissue, and the process continues over a prolonged period. During the remodeling phase, contraction and epithelialisation occurs, in which tissue structures are being organised, leading to the increase of the integrity and tensile strength of the wound [2].

Wound repair agents have demonstrated their importance towards the public’s health and health care resource expenditure as they contribute towards expediting the healing process of a wound formation [3,4]. In spite of the tremendous demand on wound repair agents in clinical use, the availability of drugs that are capable of stimulating the process of wound repair is still limited. With the demand of the market being higher than what the industry is capable of delivering, the development and utilisation of effective wound repair agents of natural origins are of great interest, not only for researchers but also to the general public and those within the healthcare industry as well.

Curcumin has always been well known for its effects in promoting wound healing [5,6,7]. However, due to its poor solubility, oxidative degradation, light sensitivity, and poor bioavailability, there has been a huge limitation in its use as an oral or even topical medication [8,9]. A vast amount of effort has been made to improve the curcumin delivery system in order to increase its stability and bioavailability to an optimum level. To improve its bioavailability and increase its permeability, several formulations have been prepared which include nanoparticles, liposomes, micelles, and phospholipid complexes [10]. For instance, nanoglobules-based nanoemulsion formulation has been prepared to enhance the solubility of curcumin [11,12]. Another formulation designed for improvement of the bioavailability of curcumin is liposomal curcumin. Liposomes are considered effective carriers to solubilise curcumin and alter the pharmacokinetic properties of curcumin. For example, liposome-encapsulated curcumin, silica-coated flexible liposomes loaded with curcumin, and curcumin-loaded flexible liposomes without silica-coatings have shown to improve the bioavailability of curcumin. In addition, liposome-curcumin formulation had been shown to improve the bioavailability of curcumin [13,14]. Cyclodextrin had also been reported for its use in curcumin formulation. In a previous publication, it was found that cyclodextrin-encapsulated curcumin had a greater cellular uptake and longer half-life in cells and showed an improvement of curcumin permeability to penetrate animal skin tissue [15,16]. Poly(lactic-*co*-glycolic acid) (PLGA) has also been used to improve the pharmacokinetics of curcumin by enhancing its bioavailability. PLGA-curcumin and PLGA-polyethylene glycol (PEG) nanoparticle-encapsulated curcumin were able to increase the half-life of curcumin through the augmentation of its bioavailability [17,18].

A topical treatment of curcumin is a medication with the application of curcumin on the skin to treat ailments such as cancer and inflammation, and to promote wound healing. The wound dressing formulation of curcumin are mainly prepared either by the use of natural polymeric materials, such as chitin, chitosan, or alginate, or with different synthetic polymers. Currently, a number of topical formulations of curcumin for their application in wound-healing have been formulated, where polymers are blended into different forms such as films, fibers, emulsion, and hydrogels, as well as with nano-formulations by the use of natural polymeric materials, such as chitin, chitosan or alginate, or with synthetic polymers [19]. For instance, poly(lactic-*co*-glycolic acid) nanoparticles encapsulating curcumin and curcumin loaded in liposomes and penetration enhancer-containing vesicles have showed their potential in improving wound healing and skin regeneration, with an enhanced level of bioavailability of curcumin [20,21]. Other delivery systems, such as biodegradable hydro gel systems have also showed potential as a great application for wound healing [22] through the controlled release of curcuminoids [23]. In this present study, a complex coacervation microencapsulation approach was used to encapsulate the curcuminoids and its wound healing-promoting effects were verified by in vitro and in vivo assays. 

## 2. Materials and Methods 

### 2.1. Materials 

Nembutal^®^ sodium pentobarbital was bought from CEVA Santé Animale, France while 6-hydroxy-2,5,7,8-tetramethylchroman-2-carboxylic acid (Trolox) was purchased from Calbiochem, Darmstadt, Germany. Phosphate buffer solution (PBS), potassium persulfate, haematoxylin, eosin, 2,2′-azino-bis(3-ethylbenzothiazoline-6-sulphonic acid) (ABTS), 2,2-diphenyl-1-picrylhydrazyl (DPPH), butylated hydroxytoluene (BHT), butylated hydroxyanisole (BHA), sodium chloride (NaCl), chloramphenicol, tetracycline, gentamicin sulphate, perchloric acid (ACS reagent, 70%), Tween 80, Span 80, Pachloramine-T-trihydrate (ACS reagent, 98%), and *n*-propanol were purchased from Sigma-Aldrich, St. Louis, MO, USA. Paraffin (tissue embedding medium, Paraplast^®^ Regular) was bought from Sigma-Aldrich, St. Louis, MO, USA. 3-(4,5-dimethylthiazol-2-yl)-2,5-diphenyl-tetrazolium bromide) (MTT) was procured from Amresco, Dublin, Ireland. 4-(dimethylamino) benzaldehyde was obtained from Fluka, Buchs, Switzerland, and liquid paraffin (extra pure) was purchased from QRëC, Selangor, Malaysia. White soft paraffin BP was bought from Euro-Pharma Sdn. Bhd., Pulau Pinang, Malaysia. Cetostearyl alcohol BP was obtained from Wilheim Wilzein Company GmbH, city, Germany. Dulbecco’s Modified Eagle’s medium (product no.: 31053-028), fetal bovine serum (FBS), penicillin and streptomycin were purchased from Gibco, Invitrogen Corporation, Waltham, MA, USA. The human keratinocyte (HaCaT) cell line and 3-(4,5-dimethylthiazolyl-2)-2,5-diphenyltetrazolium bromide) (MTT) Cell Proliferation Assay (ATCC^®^ 30-1010K^TM^) kit were obtained from the American Type Culture Collection (ATCC), Manassas, VA, US. Muller Hinton agar and Muller Hinton broth were purchased from HiMedia, India. Dimethyl sulphoxide (DMSO) and hydrochloric acid (HCl) were bought from Sigma, USA. Formaldehyde 37–41% was bought from Fisher Scientific, Loughborough, UK. The curcuminoids (mixture of curcumin, demethoxycurcumin, and bisdemethoxycurcumin, >98%) and ethanol were supplied by Acros Organics, Belgium., USA. Sodium citrate and sodium acetate were purchased from R&M Chemicals, Selangor, Malaysia. Sodium hydroxide was purchased from System, Malaysia. Silfazine cream (Sunward Pharmaceutical Sdn. Bhd., Johor, Malaysia) was obtained from a local pharmacy (Penang, Malaysia).

### 2.2. Animal Models

Healthy Sprague Dawley rats weighing 200–220 g were used for the study. The rats were kept individually in their cages and maintained with normal food and water ad libitum. The study was approved by the Animal Ethnics Committee, Universiti Sains Malaysia (Animal ethics approval no.: USM/Animal Ethics Approval/2012/(81) (426)).

### 2.3. Methods

#### 2.3.1. Complex Coacervation Microencapsulation

Gelatin B and chitosan were separately dissolved in 1% *w/w* acetic acid. The curcuminoids (0.2 g) were suspended in Tween 80, and then dispersed in 20 mL of chitosan solution using mechanical stirring at the speed of 1000 rpm at 50 °C for 30 min. After that, 20 mL of gelatin solution was added at 1 mL/min using a syringe pump (Green Stream^®^ SY-P Argus 600, ARGUS Medical AG, Heimberg, Switzerland) under constant stirring at 500 rpm with the temperature being kept constant at 50 °C (IKA^®^ Werke Staufen, Breisgau, Germany). The mixture was mixed homogeneously making the total polymer concentration 2.55% *w/w*, with the mixing ratio of gelatin to chitosan 30:1% *w/w*. Thereafter, the pH of the colloid was adjusted carefully to pH 5.50 by adding 1 M of sodium hydroxide solution (NaOH). Then, the stirring at 500 rpm was continued for another 4 h at 50 °C to induce coacervation. Following that, the liquid coacervate was gradually cooled to room temperature, then abruptly cooled to <10 °C by incubating the system in an ice bath under constant stirring for another 1 h. Subsequently, 1 mL of formaldehyde was added drop by drop into the system and stirred for 30 min to produce a covalent cross-linked microcapsule. The drug-loaded coacervate was washed with ethanol 3 times and then with cold distilled water for the final wash. This was followed by centrifugation at 1000 rpm for 5 min at a constant temperature of 10 °C. Then, the formed coacervate was frozen overnight at −70 °C followed by freeze drying (Labconco, Kansas, MO, USA). Finally, the freeze-dried microcapsules were stored in airtight glass bottles, protected from light, and kept in desiccators until required for the studies.

#### 2.3.2. Preparation of Curcuminoids Microcapsule-Incorporated Cream

The oil components (liquid paraffin, white soft paraffin, and cetostearyl alcohol of ratio 50:25:25% *w/w*), emulsifiers blend (Tween 80/Span 80), and 0.1 M citrate buffer pH 5.0 at a ratio of 30:12.95:57.05% *w/w* were mixed and prepared in a beaker. The preservatives (0.1% methyl paraben and 0.05% propyl paraben) were included in the final formulation where propyl paraben was dissolved in the oil phase and methyl paraben was dissolved in the aqueous phase. After the cream base was developed, the microcapsulated curcuminoids were dispersed and mixed in the base at 2% *w/w*. The finished cream was kept in a tightened glass jar at room temperature and protected from light prior to the experiment.

#### 2.3.3. Antioxidant Activity

##### Trolox Equivalent Antioxidant Capacity (TEAC)

The total antioxidant activity of the active ingredient(s) was estimated using the trolox (6-hydroxy-2,5,7,8-tetramethylchroman-2-carboxylic acid) equivalent antioxidant capacity (TEAC) test as described by Re et al. [24]. ABTS [2,2′-azino-*bis* (3-ehylbenzothiazoline-6-sulfonate)] was dissolved in deionized water to produce a 7 mM solution. The ABTS radical cation (ABTS**·**^+^) were produced by reacting the ABTS stock solution with 2.45 mM potassium persulfate. The mixture was allowed to stand in the dark at room temperature (24–26 °C) for 12–16 h prior to use. The ABTS**·**^+^ solution was then diluted with PBS (pH 7.4) to a final concentration that would give an absorbance of 0.70 ± 0.02 at a wavelength of 734 nm in an ambient temperature of 30 °C. The reaction was initiated by the addition of 10 µL of curcuminoids and the microcapsules of the curcuminoids solution (1 mg/mL in methanol) to 2 mL of diluted ABTS**·**^+^. The spectrophotometer (Hitachi U-2000, Tokyo, Japan) was preliminarily blanked with PBS. The decrease in absorbance was measured at 734 nm, 6 min after the addition of trolox and the samples. Trolox, a vitamin E analogue with a concentration of 0 to 4 mM, was used as the standard and for calibration purposes. BHT, a potent inhibitor of lipid peroxidation and BHA were used as the positive controls. All antioxidants were prepared in methanol and sample determinations were carried out in triplicates. The TEAC value was defined as the concentration of standard trolox with the same antioxidant capacity as a 1 mM concentration of the antioxidant compound under investigation. 

##### Assessment of DPPH Scavenging Activity

The DPPH (2,2-diphenyl-1-picrylhydrazyl) scavenging activities of native curcuminoids and the microcapsules of curcuminoids were determined according to the method in the literature [25,26]. BHT and BHA were used as references. The method was carried out by pipetting l00 mL of the samples into 96-well plates and performing a serial 2-fold dilution using methanol. Then, 200 mL of 0.2 mM DPPH solution (in methanol) was pipetted into each well and the plates were incubated at room temperature (24–26°C) for 30 min. The absorbance of the mixture was measured using a microplate reader (Power Wave X340, Winooski, VT, USA) at 517 nm against the blank (100 mL substance + 200 mL methanol). The percentage of samples’ radical scavenging activity was evaluated by comparing them with a standard (100 mL methanol + 200 mL of 0.2 mM DPPH). Each sample was measured in triplicate and the average was determined. The radical scavenging activity was calculated using the following formula:

Radical scavenging activity = [(*A*_0_ − *A*_1_)/*A*_0_] × 100

where *A*_0_ is the absorbance of the control and *A*_1_ is the absorbance of samples after 30 min. The EC_50_ (effective concentration of 50%) of the sample was determined by the equation from the dose-dependent free radical scavenging curve.

#### 2.3.4. Minimum Inhibitory Concentration

The bacterial strains used for the study were *Staphylococcus aureus* ATCC 23923, *Escherichia coli* ATCC 25922, *Klebsiella pneumoniae* ATCC 13883, *Staphylococcus epidermidis* ATCC 12228, *Pseudomonas aeruginosa* ATCC 27853, and *Bacillus subtilis* ATCC 6633. The microdilution method was used to evaluate the minimum inhibitory concentration (MIC) of curcuminoids and their effects after the microencapsulation process on both gram positive and gram negative bacteria. MIC determination was performed as reported by Luseba et al. with some modification [27]. The colony of bacteria (3–5 colonies) was sub-cultured in 5 mL of sterile Mueller Hinton Broth (MHB) and incubated (Incubator shaker series PSE-T150, Stik^®^ Instrument Equipment, Shanghai) for 16–20 h at 37 °C. For bacteria such as *P. aeruginosa, S. aureus*, and *S. epidermidis*, the MHB was supplemented with 2% sodium chloride. Then, 100 µL of the bacterial suspension was transferred to 10 mL of freshly prepared, sterile MHB or MHB supplemented with sterile 2% NaCl according to the type of bacteria. The new bacterial suspension was then incubated in an incubator shaker at 37 °C for 2–3 h. The turbidity of the inoculum was checked using a spectrometer (Thermo Spectronic, model 4001/4, USA) at 600 nm to ensure that the optical density (OD) obtained was 0.002. The stock solutions of the curcuminoids and curcuminoids microcapsules were prepared in dimethyl sulfoxide (DMSO) with 5% DMSO as the final concentration in the microplate. Gentamicin, tetracyclin, and chloramphenicol were used as positive controls. These antibacterial agents were then transferred in volumes of 100 µL into a sterile 96-wells plate from column A to G. Then, a 2-fold serial dilution was conducted using MHB, or MHB supplemented with 2% NaCl when bacteria *P. aeruginosa*, *S. aureus*, and *S. epidermidis* were the bacteria tested. The wells in the last column of the plate (column H) served as a drug-free control that contained only the broth without any drugs or microbes. Subsequently, 100 µL of bacterial inoculum was transferred into all of the 96 wells, and the final concentration of the bacterial suspension was ~5 × 10^5^ CFU/mL. The final inoculum was used within 30 min as an increase of bacterial density occurs over time. Thereafter, the plate was covered and incubated in an incubator at 37 °C for the next 16–20 h. Then, 50 µL of 0.2 mg/mL MTT [3-(4,5-dimethylthiazol-2-yl)-2,5-diphenyl-tetrazolium bromide] was added to each of the wells and the plate was incubated in the incubator at 37 °C for 30 min. The reduction in the purple colour indicated bacterial growth inhibition, whereas clear wells indicated a lethal effect of the samples on the bacteria. The substances were tested in triplicates to obtain the average.

#### 2.3.5. Effects of Curcuminoids and their Encapsulated Form on HaCaT Cell 

##### Cell Culture and Treatments

The human keratinocyte (HaCaT) cell line was obtained from the American Type Culture Collection and cultured in Dulbecco’s Modified Eagle’s Medium supplemented with 10% fetal bovine serum (FBS), penicillin (100 IU/mL), and streptomycin (100 µg/mL). The cell line was maintained in an incubator set at 37 °C with a constant supply of 5% carbon dioxide. 

HaCaT cells were then plated when the cells reached 70% confluency. After overnight incubation, the cells were subsequently treated with varying concentrations (ranging from 0.1–100 μM) of either curcuminoids or curcuminoids microcapsule. The test compounds were dissolved in DMSO and diluted using the culture medium before being introduced to the cells.

##### Cell Cytotoxicity/Viability Determination by 3-(4,5-dimethylthiazol-2-yl)-2,5 Diphenyltetrazolium Bromide (MTT) Assay

For the determination of cell cytotoxicity, HaCaT cells were seeded into a 96-well plate at a density of 3000 cells per well. Each well is filled with the test compounds to a final volume of 100 µL/well. The cells were then incubated with various treatments for 24, 48, and 96 h. A total of 10 µL of 5 mg/mL MTT were then added into each wells, making the final concentration 0.45 mg/mL and the plate was incubated for 4 h. A total of 100 µL of dimethyl sulfoxide (DMSO) was added and mixed well, and then the absorbance was read at 570 nm at different intervals. Viable cells with active metabolism will cause a conversion of MTT into a purple-coloured formazan product with a maximum absorbance near 570 nm. DMSO was then added to dissolve the formazan crystals formed. The percentage of cell viability compared to the control was calculated in which the untreated cells served as the control. 

##### Cell Proliferation Determination by 3-(4,5-dimethylthiazol-2-yl)-2,5-diphenyltetrazolium Bromide (MTT) Assay

For the determination of cell proliferation, HaCaT cells were seeded into a 24-well plate at a density of 10,000 cells per well. A total of 500 µL of test compounds were then added to each of the wells and the cells were cultured for 24–96 h at 37 °C with a constant supply of 5% CO_2_ in a humidified incubator. A MTT assay was then performed and the absorbance was taken every day for 4 days to monitor the rate of cell proliferation. A total of 50 µL of MTT (5 mg/mL) reagent was added to each of the wells and incubated at 37 °C and 5% CO_2_ for 3–4 h until a purple precipitate was visibly formed. Then, 500 µL of Detergent Reagent was added to each of the wells and the plate was left in the dark at room temperature for 2 h to dissolve the formazan crystals. After that, the absorbance was read at 570 nm using the Infinite 200 PRO microplate reader (Tecan, Männedorf, Switzerland). 

##### Cell Migration Determination by Wound Scratch Assay

HaCaT cells were seeded into a 6-well plate at a density of 2 × 10^5^ cells per well. Scratch lines were made using a 1000 μL pipette tip. Test samples of 1 mL was then added into each well. The scratch lines were observed under an inverted microscope equipped with a camera (Nikon, Melville, NY, USA) and the images were captured. The width of the lines was measured using the NIS-Elements D Microscope Imaging Software (Nikon, USA) at 24, 48, and 96 h of treatment. The percentage of gap closure was calculated using the following formula:

$$\mathrm{Gap}\;\mathrm{closure}\;(\%)\;=\;\frac{\mathrm{Average}\;\mathrm{distance}\;\mathrm{of}\;\mathrm{gap}\;\mathrm{at}\;0\;h\;-\;\mathrm{Average}\;\mathrm{distance}\;\mathrm{of}\;\mathrm{gap}\;\mathrm{at}\;x\;\mathrm{hour}}{\mathrm{Average}\;\mathrm{distance}\;\mathrm{of}\;\mathrm{gap}\;\mathrm{at}\;0\;h}\;\times \;100$$

#### 2.3.6. In Vivo Wound Healing Study of Curcuminoids Microcapsule-Incorporated Cream

Healthy Sprague Dawley rats were anaesthetised using pentobarbital (60 mg/kg, IP) and the fur on the back and flanks on both the left and right sides were shaved with a sterile standard electrical shaving machine. The shaved areas were then disinfected with 70% *v/v* ethanol. Following that, a modified stainless steel stamp (20 mm in diameter) with an electronic temperature controller and a thermocouple type feedback sensor that has been heated to 130 °C was applied on the skin of the subjects between the twelfth rib and the horizontal upper limits of the sacroiliac joints for 5 s to produce a second-degree burn [28,29]. The animals were then returned to their cages with the tether positioned well out of reach of the animals.

Following the burn infliction procedure, 96 animals were randomly divided into 4 groups with 18 rats in each group. Each group’s animals were evaluated at 4 different sampling times at 0, 7, 14, and 21 days (6 rats in each group for each sampling time). Each group of rats were tested using different samples with Group 1 being treated with the cream base to act as the normal control group, while the rats from Group 2 were treated with sterile normal saline as the negative control. Group 3 rats were treated with 0.5 g Silfazine cream (silver sulphadiazine 1% *w/w*) as the positive control [30], and the rats from Group 4 were treated with 0.5 g of 2% curcuminoids microcapsule cream. The tropical treatments were applied once daily up to 21 days post-burn induction. Throughout the entire period of treatment introduction, the wound contraction rate was measured and represented as the reduction in wound size on day 3, 6, 9, 12, 15, 18, and 21. The progressive decrease in the wound size was monitored periodically by tracing the boundary of the wound using a digital calliper. For morphological analysis, the animals were euthanised via carbon dioxide inhalation according to the “IACUC Guideline—Carbon Dioxide Euthanasia of Rodents” [31] and fragments of the affected skin were collected from the subjects of each group at 7, 14, and 21 days after burn induction for histopathological and hydroxyproline evaluations. 

##### Measurement of Hydroxyproline

On day 21 post-burn induction, a piece of skin from the healed wound area was collected and analysed for its hydroxyproline content. The assay was performed following the protocol published by Woessner cited in Jorge et al. [32]. A total of 30 g of tissues were excised and dried in a hot air oven at 60–70 °C to obtain their constant weight, and then the tissue were hydrolysed in 1 mL of 6 N HCl at 130 °C for 4 h in sealed glass tubes. The hydrolysate was neutralised with 1 mL of 2.5 N NaOH. Then, 20 µL of each of the hydrolyzed samples were added to the 96-well plate and incubated for 20 min at room temperature with 50 µL/well of chloramines T solution (282 mg chloramines T, 2 mL *n*-propanol, 2 mL distilled water, and 16 mL citrate acetate buffer). Next, 50 µL/well of Erlich’s solution (2.5 g of 4-(dimethylamino)benzaldehyde, 9.3 mL of n-propanol, and 3.9 mL of 70% perchloric acid) was added and incubated for 15 min at 65 °C. The absorbance was measured at 550 nm using a microplate reader (Power Wave X340, USA). Hydroxyproline concentrations from 0 to 10 µg/mL were used to prepare a standard curve, and the results were expressed as µg/mL of hydroxyproline.

##### Histopathologic Study

The fragments of skin collected from the animals of each group at 7, 14, and 21 days following burn induction were investigated for histopathological evaluation. Sample tissues were fixed in 10% formalin and processed by the dehydration and clearing of the samples using an automatic tissue processor (model Citadel 1000, Shandon, Cheshire, UK). After processing, the tissues were then embedded in paraffin with a Histo-Center II-N (Barnstead/Thermolyne Corp., Dubuque, IA, USA) and sectioned to a thickness of 5 µm using a Histocut 820 (Reichert-Jung, Nussloch, Germany). The tissues were stained using haematoxylin and eosin, and later examined using a light microscope to observe ulceration, necrosis, and epithelialisation of the skin tissues.

### 2.4. Statistical Analysis

The data were expressed as mean ± S.D. or S.E.M. one-way analysis of variance (ANOVA) followed by the Dunnett Multiple Comparison Test, were used to compare the treated groups with the control group by SPSS (statistical package for social sciences) version 17.0.

## 3. Results

### 3.1. Antioxidant Activity

The total antioxidant activity was expressed as mM trolox equivalent. The higher the mM trolox equivalent values, the more potent the samples are in terms of their antioxidant activities. The TEAC and EC_50_ values of DPPH scavenging activity of the curcuminoids and curcuminoids microcapsule are presented in Table 1. The results showed that microencapsulation does not influence the antioxidant capacity of curcuminoids. This study also demonstrated that the antioxidant activity and DPPH scavenging effect of curcuminoids and curcuminoids microcapsule were much higher compared to BHT and BHA.

### 3.2. Minimum Inhibitory Concentration

The minimum inhibitory concentrations of the samples are shown in Table 2. The results demonstrated that the active ingredients being tested and the antibiotics exhibited varying degrees of MIC values against the studied microorganisms. The MIC values of the curcuminoids microcapsule range from 16 to 64 µg/mL. The results also indicated that the antibacterial activity of the curcuminoids microcapsule was comparatively greater for *S. aureus*, *E. coli*, and *S. epidermidis* than its un-encapsulated form. 

### 3.3. Effects of Curcuminoids and the Curcuminoids Microcapsule on HaCaT Cell

#### 3.3.1. Cell Cytotoxicity/Viability Determination by 3-(4,5-dimethylthiazol-2-yl)-2,5 Diphenyltetrazolium Bromide (MTT) Assay

**Figure 1:**
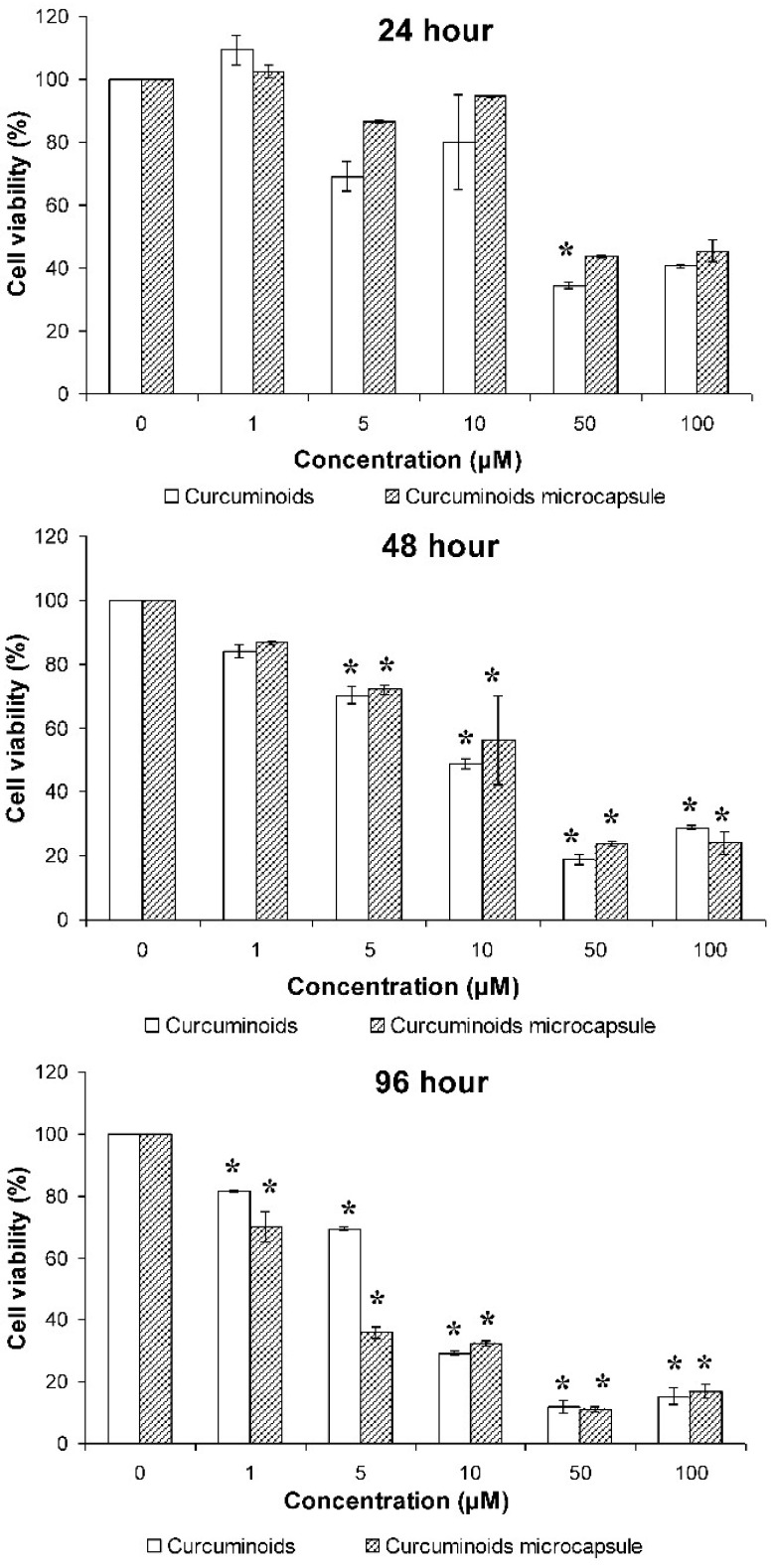
Effects of the native curcuminoids and curcuminoids microcapsule at various concentrations on human keratinocyte (HaCaT) cell viability. Results were expressed as mean ± S.D. of three independent tests. * indicates *p* < 0.05 compared to the untreated control, # indicates *p* < 0.05 compared to curcuminoids group.

The effects of native curcuminoids and curcuminoids microcapsules on cell viability was determined using the MTT assay. Results showed that the curcuminoids and curcuminoids microcapsules reduced cell viability in a dose-dependent manner. Generally, the compound was not toxic to the cells when being introduced for a short duration, but exerted significant toxicity when the incubation time was being prolonged. Significant reductions in cell viability was observed when the cells were treated with these compounds for 48 and 96 h. The results also exhibited that concentrations higher than 5 μM induce cell death at 48 h. Significant cell death was noted at 96 h of incubation, even when the samples are at their lowest concentration (1 μM). In addition to that, the curcuminoids microcapsule showed more toxicity against HaCaT cells at 1 and 5 μM compared to the native curcuminoids (Figure 1). 

#### 3.3.2. Cell Proliferation Determination by 3-(4,5-dimethylthiazol-2-yl)-2,5 Diphenyltetrazolium Bromide (MTT) Assay

**Figure 2:**
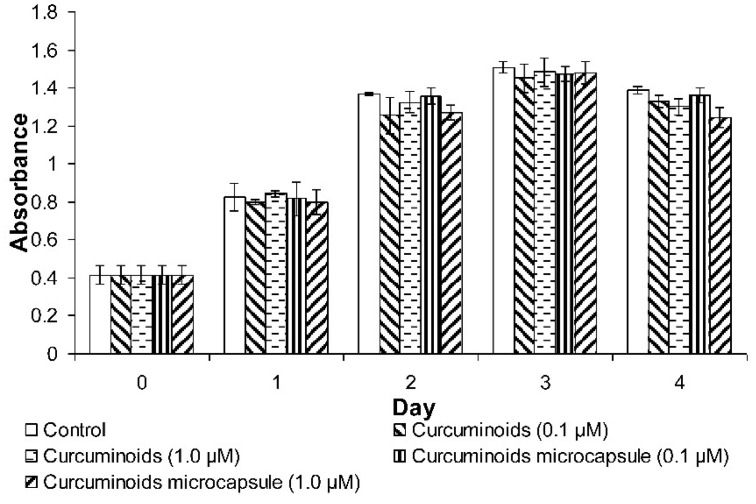
Effects of the native curcuminoids and curcuminoids microcapsule on HaCaT cell proliferation. Each point represents the mean of three independent tests. Results were expressed as mean ± S.D. of three independent tests.

The results showed increments in cell number of the control and treated groups over a 4 day period. However, no significant differences were observed between the treated cells and those from the untreated control (Figure 2).

#### 3.3.3. Cell Migration Determination by Wound Scratch Assay

**Figure 3:**
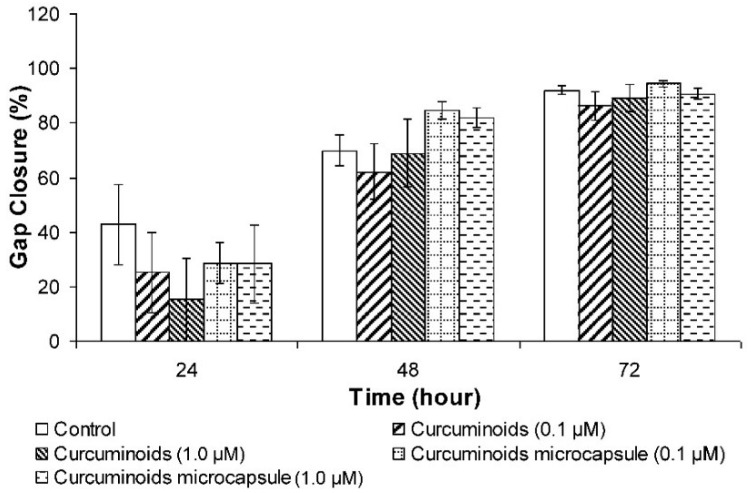
Effects of native the curcuminoids and curcuminoids microcapsule on the scratch wound repair of HaCaT cells. Results were expressed as mean ± S.D. of three independent tests.

The wound scratch assay was used to measure the migration of cells. At hour 24, the group that was treated showed lesser cell migration than the control group. At hour 48, the cells treated with the curcuminoids microcapsule showed a better effect in improving cell migration at 0.1 and 1 μM, compared to the control. On the other hand, native curcuminoids reduced cell migration at 0.1 and 1 μM compared to the control (Figure 3). 

### 3.4. Effects of Curcuminoids and Microcapsule Incorporated Creams as Controlled Drug Delivery on Wound Healing

#### 3.4.1. Wound Contraction

**Figure 4:**
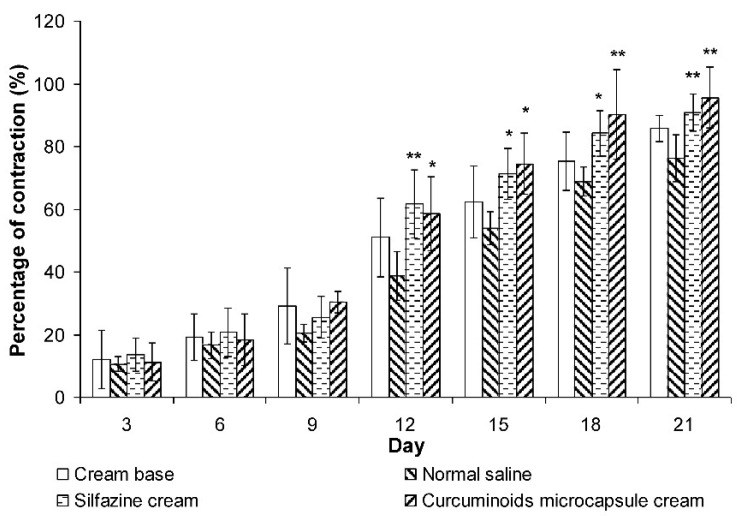
Effects of cream base, silfazine cream (positive control), and curcuminoid microcapsule cream on wound contraction compared to normal saline negative control (*n* = 6). Results were expressed as mean ± S.E.M. ** indicate significant difference at *p* < 0.05 and *p* < 0.01 compared to normal saline-treated group.

The size of the wound inflicted was measured on day 1, 3, 6, 9, 12, 15, 18, and 21 post-burn injury in all of the groups (Figure 4). Generally, the closure of wounds for all the treated and control groups gradually began from day 12 to day 21 post injury. The negative control group (treated with normal saline) showed the least contraction of wound size amongst all groups. The curcuminoids microcapsule cream treated group demonstrated significant wound closure starting from day 12 to day 21 post injury. The silfazine cream group showed significant (*p* < 0.05) wound contraction starting from day 12 to day 15. On day 21, the percentage of wound contraction for the curcuminoids microcapsule and silfazine cream treated groups were significantly different at *p* < 0.01, compared to the normal saline negative control group. This indicated that the drug-treated groups displayed a positively improved wound healing process. 

#### 3.4.2. Measurement of Hydroxyproline 

**Figure 5:**
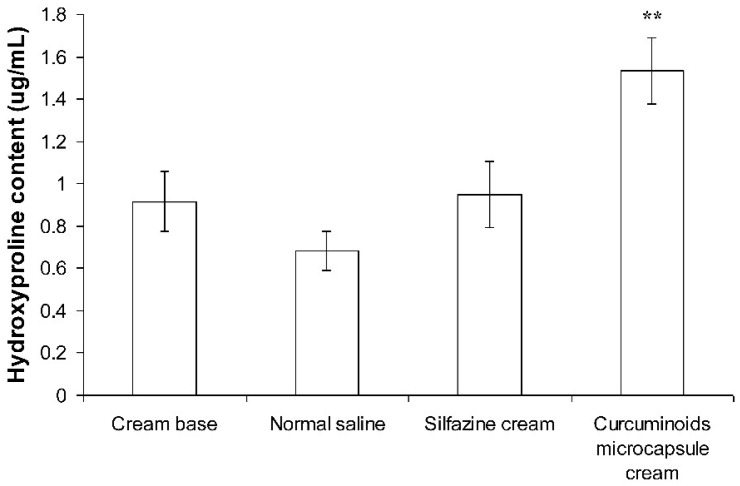
Effects of cream base, silfazine cream (positive control), and curcuminoids microcapsule cream on hydroxyproline content in wound tissue compared to the normal saline negative control group (*n* = 6). Results were expressed as mean ± S.D. ** indicate significance at *p* < 0.01, compared to normal saline-treated group.

Figure 5 demonstrates the effect of silfazine and the curcuminoids microcapsule cream on the hydroxyproline synthesis of burn wounds. The results demonstrated that the curcuminoids microcapsule cream significantly stimulated hydroxyproline synthesis (*p* < 0.01) in burn wounds. On day 21, the group with the subjects treated with the curcuminoids microcapsule cream showed 124.4% more hydroxyproline in their wound tissues than those of the negative control group treated with normal saline. On the other hand, the silfazine cream, and the cream base treated groups did not show any significant increase in hydroxyproline synthesis.

#### 3.4.3. Histopathologic Study 

**Figure 6:**
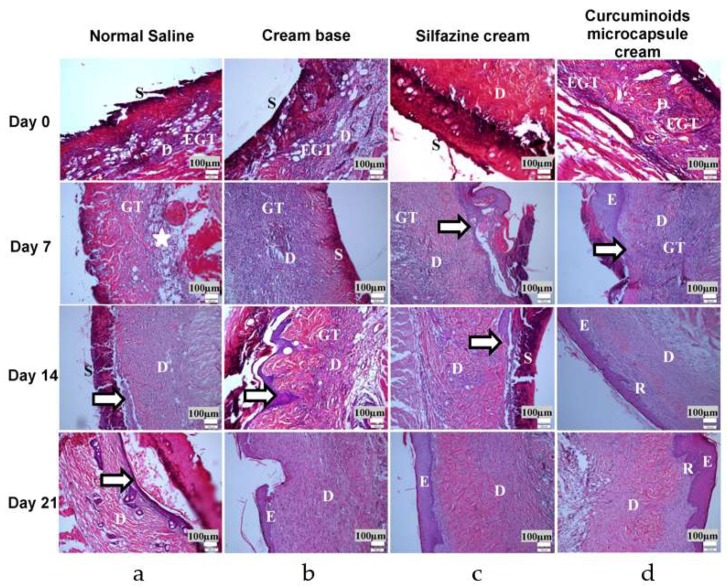
Hematoxylin and eosin stained sections of skin burn wound from: (**a**) Normal saline (negative control), (**b**) cream base-treated, (**c**) silfazine cream-treated, and (**d**) curcuminoids microcapsule cream-treated; (E) epidermis, (S) scab, (D) dermis, (EGT) early granulation tissue, (GT) granulation tissue, (star) aggregation of inflammatory cells, (R) rete ridges, and (→) epithelialisation.

The histological findings on day 0 (within 24 h post injury) in all five groups showed similar characteristics in terms of skin damage (Figure 6). All the burns inflicted can be safely categorised as second degree in all subjects as macroscopic analysis found that the upper third to half of the dermis was damaged and injured during the burn wound induction. Most of the epidermal layers and basement membrane were shown to be disrupted. On day 7 post injury, both groups treated with the curcuminoids microcapsule cream and silfazine cream produced an accelerated degree of epithelialisation on the wound sites, with the curcuminoids microcapsule cream-treated group showcasing a more significant increase in terms of epithelialisation level, with an initiation of the reestablishment of the basement membrane. There were also less inflammatory cells found in these groups in contrast with the negative control (normal saline) and cream base groups which had a moderate invasion of inflammatory cells in the damaged region and no observable epithelialisation. On day 14 post injury, epithelialisation was similarly active in all groups, with the basement membrane starting to develop in the curcuminoids microcapsule cream, silfazine cream, and cream base-treated groups. On the other hand, only the subjects receiving the curcuminoids microcapsule cream showed a regenerated dermal-epidermal junction, with extensive formations of rete ridges and capillary loops. However, no formations of basement membrane can be identified in the negative control group. On day 21 post injury, complete epithelialisation was observed in all of the groups except for the negative control. 

## 4. Discussion

The total antioxidant activity demonstrated by curcuminoids was due to its phenolic hydroxyl group [33]. A higher TEAC value indicates greater antioxidant activity in the scavenging of the free radical cation 2,2′-azino-*bis* (3-ehylbenzothiazoline-6-sulfonate) (ABTS**·**^+^). Antioxidants act as hydrogen donors and terminate the oxidation process of free radicals by converting them to more stable products [34].

The effects of curcuminoids and quercetin on DPPH radical scavenging is due to their hydroxyl groups which scavenge the free radicals via hydrogen donation. This causes a decrease in the absorbance of the DPPH radical, which is a stable free radical that accepts an electron on its hydrogen ion, thus converting into a stable diamagnetic molecule [35]. When this electron is paired off, the absorption decreases stoichiometrically with respect to the number of electrons taken up [36]. Therefore, the reduction of DPPH is proportional to the decrease of absorbance at 517 nm. 

The cytotoxic effect of curcuminoids and its microencapsulated form on keratinocytes, the HaCaT cells, was investigated. There was negligible cell death when the HaCaT cells were exposed to the compound for 24 h. However, when the incubation time was increased, the compounds showed noticeable cell toxicity, in which a significant drop in cell viability was noted in almost all of the concentrations of compounds tested even at the lowest concentration (1 μM). Previous literature appears to be inconsistent with the cytotoxic effect of curcumin shown in this study, particularly in the cell culture assays. Banerjee et al. found that curcumin acted as an antioxidant at low concentrations but the mode of mechanism of action changed to prooxidant at high concentrations (>10 μM) [37]. The cell-protecting properties of curcuminoids may come from their antioxidant effect, while their prooxidant effect may have contributed to their cell cytotoxicity. This prooxidant property may be the cause of the significant cell death that was observed in the current study, which is supported by a number of studies discussing the toxic effect shown by curcuminoids on cells [37,38]. 

It is interesting to note that the microencapsulated form of the curcuminoids was more toxic than its non-encapsulated counterpart. This may be due to the drug-release control features of encapsulation (t50 for curcuminoids and curcuminoids microcapsule were 22.75 and 17.18 h, respectively) [39]. Cells were continuously exposed to the compound at a steady rate, making the effect of the curcuminoids a whole lot more enhanced and stronger than the non-encapsulated form. Based on current observations, the curcuminoids were safe to use for a short duration on skin cells at low concentrations. 

Re-epithelialisation of the skin is a process which is predominated by keratinocytes proliferation and migration, an essential event in wound healing. The results showed that neither the curcuminoids nor the microencapsulated curcuminoids improved HaCaT cell proliferation. On the other hand, in the wound scratch study, the microencapsulated curcuminoids showed a better effect than the non-encapsulated form in the induction of cell migration. However, the data was not statistically significant, thus it was determined that curcuminoids did not increase cell proliferation and migration. These findings are consistent with the observations from an earlier study, in which curcumin was shown to have no effect on fibroblast motility [40]. Other researches have even exhibited that curcumin inhibits cell proliferation and proposed that the inhibition is mediated through the regulation of prohibitin (PHB) in the HaCaT cells [41,42,43].

In summary, there is no evidence supporting the effectiveness of curcuminoids and their encapsulated form in terms of promoting wound healing in vitro in the present study. However, the effect of the compounds should be tested in in vivo models as wound healing is a complex process, involving interactions between the cells’ intracellular and extracellular matrix and is affected by various systems within an organism [44]. The wound healing process involves inflammation, granulation tissue formation, epithelialisation, and new tissue remodeling to reconstruct and restore damaged tissues to theirs near normal and optimal state [45]. During the process, connective tissue repair, re-epithelialisation, and angiogenesis is required. Various literature has shown that a number of mechanisms influences the healing rate, such as the modulation of inflammatory mediators [37]. 

The microencapsulated curcuminoids showed slightly improved antioxidant and in vitro antibacterial activities compared to their non-encapsulated forms, similar to the results from Suwannateep et al. who had found comparable antioxidant activities between the encapsulated and non-encapsulated versions of curcumin [46]. From our results, it was shown that the microencapsulated curcuminoids exhibited controlled drug release (zero order kinetics), increased photo-stability (kept in a desiccator at room temperature (28 ± 4 °C/75 ± 10% RH), and was allowed to receive sunlight exposure for one month. The rate constants for the samples were obtained from their respective best-fitted kinetic plots and used to calculate the half-life degradation (*t*_1/2_) of the studied samples and found that t_1/2_ of microencapsulated curcuminoids up to 236 days as compared to free curcuminoids (*t*_1/2_ of 66.63 days), non-staining, and extended shelf-life (kept in long-term storage condition (28 ± 4/75 ± 10% RH for 12 months, 40 ± 2 °C/75 ± 5% RH, and 5 ± 3 °C, for 6 months). CPM showed excellent chemical stability during storage periods due to the curcuminoids being protected by the capsule shell from adverse environment conditions. Suwannateep and colleagues reported in their studies that encapsulation increased the photostability of curcumin nanoparticles and therefore prolonged the antioxidant activity of curcumin compared to free curcumin in lotion formulations. This result parallels those discussed regarding the stability of microencapsulated curcuminoids. Further supporting evidence of this theory was shown in previous researches, in which findings portrayed how the free curcuminoids have a tendency to become unstable after being exposed to light or heat, as well as losing their activity during storage [47,48,49]. This finding is especially important when the active ingredients are to be applied to the skin using a cosmetic or pharmaceutical formulation. Therefore, encapsulated curcuminoids cream is expected to exhibit greater activity due to its prolonged bioavailability after each topical application and increased photo-stability compared to free curcuminoids. Thus, based on the above reasons, the animal study of burn wounds was designed to focus on the microencapsulated curcuminoids in the topical cream formulation and their effectiveness on wound healing was compared with the marketed and typically recommended silver sulfadiazine cream. 

Animal models are commonly used in wound healing research that is not feasible with human models, such as burns, dehiscence, ulceration, infection, and scarring [50]. The use of an in vivo model is inevitable in wound healing studies, and using animal models enable the induction of wounds that are similar in consistency in terms of the size, shape, and severity of the injury, facilitating acceptable comparisons of data between studies. Moreover, the wound healing process of animals is of an accelerated period, making it possible to study the entire wound healing process in days rather than the weeks as required by humans for the complete healing of a wound [51]. The wound healing study was conducted for 21 days because this duration is crucial in obtaining the final results of wound healing [28,52].

However, there are significant anatomical and physiological differences between human and rat skin that should be noted. Rats have loose skin and contraction is one of the major features of healing in wounds inflicted on them [45]. Wound contraction is a fibroblast-dependant process and involves the deposition and maturation of collagen, the predominant extracellular protein in the granulation tissue of the wounds [53]. It is also a principal component of connective tissues, which plays a role in the healing of wounds and providing a structural framework for regenerating tissues [44]. The role of collagen in wound healing commences almost immediately upon wound formation as there is a significant hike in the synthesis of collagen around the injury site. This synthesis does not stop for months after the wound appears to be healed. Collagen plays a role in haemostasis and in providing both strength and integrity to the wound matrix and epithelialisation. Since hydroxyproline is a major component of the collagen protein, an estimation of hydroxyproline present is an accepted method to biochemically evaluate the total collagen content of a sample. It is also used as a marker for collagen synthesis [54]. Hence, an increase in collagen may be attributed to an increase in collagen synthesis or an increase in the proliferation of fibroblasts that synthesise collagen. Several research articles have also reported that the progress of wound healing correlates with the amount of hydroxyproline contained within a wound [54].

Curcuminoid containing herbs have been used for the management of burns and skin wounds in traditional medicinal practices. In the present study, the application of the microencapsulated formulation as a delivery system for curcuminoids as a topical medication produces a significantly enhanced rate of wound healing, as shown in the measurement of wound contraction and in histological studies. The data revealed that the curcuminoids microcapsule cream significantly enhanced collagen formation via the measuring of hydroxyproline (a marker of collagen synthesis) content in the wounds. Additionally, the histological score demonstrated that the curcuminoids microcapsule cream-treated group had a more rapid epithelialisation rate compared to the negative control group.

Wound healing is a complex process which involves various phases, namely, haemostasis, inflammation, proliferation, and remodeling. Among these phases, inflammation is the most prominent phase, where uncontrolled inflammatory responses will lead to the over-production of reactive oxygen species (ROS) resulting in oxidative damage that inhibit the wound healing process. It has also been reported that antioxidants with free radical scavenging activity can improve the wound healing process [3]. The present study clearly provided evidence that curcuminoids acts as excellent antioxidants with the relative antioxidant effect of curcuminoids microcapsule showing to be enhanced compared to the non-encapsulated forms. This suggested that the antioxidant property of curcuminoids may play an important role in the wound healing process.

As mentioned above, the proliferative phase (which includes fibroblast proliferation and epithelialisation) is also a crucial phase in wound healing. The infiltration of fibroblasts into wounds will promote tissue formation, collagen production, and collagen deposition [55]. Studies have shown that the migration and proliferation of fibroblast to the wound site ensured a good progression of the wound healing process [56]. In the wound, fibroblast will differentiate into myofibroblasts to augment the tissue matrix. Research have shown that there is also interaction of keratinocyte and fibroblast, whereby keratinocytes initiates growth factors in fibroblasts, which themselves stimulate keratinocyte proliferation. However, the in vitro study showed that the non-encapsulated curcuminoids, as well as the microencapsulated curcuminoids had no significant effect on HaCaT cell proliferation and their migration kinematic. These contradictory results indicated that in vitro wound healing models are still inadequate at mimicking the truly complex nature of the wound healing process that occurs in the in vivo settings [40]. These findings also agreed with previous studies in which curcuminoids caused keratinocyte apoptosis in in vitro wound models [57]. Therefore, the in vitro test results should not represent the in vivo test results as they may contradict each other. 

## 5. Conclusions

The curcuminoids microcapsule incorporated cream demonstrated an increase in the rate of wound contraction and lead to better epithelialisation rates. Hence, curcuminoids microcapsule incorporated cream would be highly beneficial for the wound healing process and recommended for effective topical wound healing therapy.

## Figures and Tables

**Table 1:** Antioxidant activity of curcuminoids, curcuminoids microcapsule, butylated hydroxyanisole, and butylated hydroxytoluene.

Compounds	* TEAC (mM) of 1 mg/mL Tested Compound (mean ± S.D., *n* = 3)	DPPH Scavenging EC_50_ (µg/mL) (mean ± S.D., *n* = 3)
Native curcuminoids	10.49 ± 1.19	35.55 ± 0.36
Curcuminoids microcapsule	11.17 ± 1.12	35.14 ± 1.14
BHA	3.82 ± 2.30	44.30 ± 3.21
BHT	2.23 ± 0.40	59.77 ± 2.41

Notes. * The value is relative to the antioxidant activity of 1 mM Trolox. BHA: butylated hydroxyanisole, BHT: butylated hydroxytoluene, TEAC: trolox equivalent antioxidant capacity, 2,2-diphenyl-1-picrylhydrazyl: DPPH.

**Table 2:** Minimum inhibitory concentrations of curcuminoids, microcapsule curcuminoids, and antibiotics against the gram positive and gram negative bacteria.

Drug/Antibiotic	Minimum Inhibitory Concentration (µg/mL)					
	*E. coli*	*S. aureus*	*K. pneumoniae*	*S. epidermidis*	*P. aeruginosa*	*B. subtilis*
Native curcuminoids	64	64	64	32	32	64
Microcapsule Curcuminoids	32	16	64	16	32	64
Gentamicin	0.63	2.5	>5	5	1.25	0.08
Tetracyclin	0.47	0.12	1.88	0.12	0.12	0.12
Chloramphenicol	2.19	4.38	8.75	2.19	2.19	2.19
